# Cardiac gating calibration by the Septal Scout for magnetic resonance coronary angiography

**DOI:** 10.1186/1532-429X-16-12

**Published:** 2014-01-24

**Authors:** Garry Liu, Graham A Wright

**Affiliations:** 1 Department of Medical Biophysics, University of Toronto, Toronto, ON, Canada

**Keywords:** Cardiac gating, Magnetic resonance coronary angiography, Septal scout

## Abstract

**Background:**

Electrocardiogram (ECG) gating is commonly used to synchronize imaging windows to diastasis periods over multiple heartbeats in magnetic resonance (MR) coronary angiography. Calibration of the ECG gating parameters is typically based on a cine cardiovascular MR (CMR) video of the beating heart. Insufficient temporal resolution in the cine-CMR method, however, may produce gating errors and motion artifacts.

It was previously shown that tissue Doppler echocardiography (TDE) can identify accurate diastasis window timings by observing the movement of the interventricular septum (IVS). We present a new CMR technique, the Septal Scout, for measuring IVS motion. We demonstrate that cardiac gating windows determined by the Septal Scout produce sharper coronary MR angiography images than windows determined by cine-CMR.

**Methods:**

9 healthy volunteers were scanned on a GE Optima 450w 1.5T MR system. Cine-CMR was acquired and used to identify the start and end times of the diastasis window (*W*_cine_).

The Septal Scout employs a one-dimensional steady-state free precession (SSFP) readout along the ventricular septum prescribed from the 4-chamber view. The Septal Scout data is processed to produce a septal velocity function, from which the diastasis window was determined (*W*_sep_).

Non-contrast-enhanced MR angiography was performed twice for each volunteer: once gated to *W*_cine_, once to *W*_sep_. Vessel sharpness was assessed subjectively by two experienced observers, and quantitatively by full width half maximum (FWHM) measurements of cross-sectional vessel profiles.

In addition, TDE was performed on a subcohort of 6 volunteers where diastasis windows (*W*_TDE_) were determined from the IVS velocity measured in the 4-chamber view. *W*_*sep*_ and *W*_*TDE*_ were compared using Pearson’s correlation.

**Results:**

MRA acquisitions were successful in all volunteers. Vessel segments produced smaller FWHM measurements and were deemed sharper when imaged during the Septal Scout gating windows (*p* < 0.05). Subjective assessment of sharpness also improved for the Septal Scout-gated scans (*p* < 0.01 for both observers). Lastly, *W*_*sep*_ and *W*_*TDE*_ were highly correlated (*R* > 0.98, *p* < 0.001).

**Conclusions:**

The MR Septal Scout technique was introduced and demonstrated to be more accurate at determining cardiac gating windows than cine-CMR, yielding sharper coronary MR angiography images.

## Background

Magnetic resonance coronary angiography (MRCA) is a potential diagnostic tool for coronary artery disease (CAD). Compared to the current gold standard, x-ray angiography, benefits of MRCA include three-dimensional visualization of coronary vessel lumens, and not subjecting patients to the risks associated with catheterization [1,2] and ionizing radiation [3]. MRCA, however, requires long acquisition times that span multiple heartbeats. Cardiac motion is particularly problematic for MRCA because high spatial resolution is required for diagnosing CAD.

Prospective cardiac gating remains the most effective tool for reducing cardiac motion artifacts in MRCA. The general principle behind cardiac gating is to synchronize, for each heartbeat, the imaging window with the diastasis period. Shechter et al. and Johnson et al. have measured coronary artery velocities during diastasis to be on the order of 10 mm/s [4,5]. Several authors have also estimated imaging window durations within diastasis that will limit blur artifacts to within sub-millimetre pixels. Their estimates include: 66 to 330 ms with a mean of 187 ms for the entire coronary tree [4]; 66 to 220 ms with a mean of 120 ms for the right coronary artery (RCA) [6]; and, 65 ± 42 ms for the mid-RCA [7].

Cardiac gating is most commonly facilitated by the use of (1) the electrocardiogram (ECG) for monitoring ventricular systole onset, and (2) a cine cardiovascular MR (CMR) video of a heartbeat obtained prior to the MRCA scan for finding the timing of diastasis relative to the ECG. Yet, the use of cine-CMR to determine ventricular diastasis may produce cardiac gating errors due to insufficient temporal resolution of the cine-CMR method.

The temporal resolution of a conventional cine acquisition depends on the number of *k*-space lines acquired per segment (of *k*-space) per heart beat, herein denoted lines per segment (LPS), multiplied by the acquisition time of each *k-*space line, commonly known as the repetition time (TR). The number of segments used to complete *k*-space coverage dictates the number of heartbeats required for the total scan duration. For example, consider the cine parameters: heartbeat duration, 960 ms; field of view (FOV), 35 cm; number of frames, 30; TR, 4 ms; partial Fourier acquisition matrix size, 160 × 256; and LPS, 16. The cine acquisition would span 10 heartbeats, have a true temporal resolution of 64 ms. Extending this example with approximate motion parameters, a mid right coronary artery moving at a peak velocity of 12 cm/s during rapid filling and decelerating constantly over 100 ms toward rest during diastasis will traverse 1.5 mm in the 50 ms preceding diastasis. The reverse motion may be observed transitioning from diastasis to atrial contraction. Therefore, during transitional cardiac phases pertinent to ECG gating, the conventional cine acquisition may not provide the sufficient frame rate to resolve the motion of an RCA segment. In addition to inadequate temporal resolution, cine acquisitions are susceptible to temporal data-mixing if the subject’s heart rate varies during the scan duration.

In this paper, we present a new CMR technique, the Septal Scout, for determining the timing of the diastasis period. The Septal Scout measures ventricular septal motion. We demonstrate that cardiac gating windows determined by the Septal Scout produce sharper MRCA images than windows determined by cine-CMR. To facilitate a user-independent assessment of the diastasis period from cine-CMR data, an empirical approach was chosen based on identifying a plateau period of high frame-to-frame correlations during diastole [8].

## Methods

### Study subjects

This study was approved by the Research Ethics Board at Sunnybrook Health Sciences Centre, and all volunteer subjects provided written informed consent prior to participation in the study. We studied 9 healthy male volunteers of age 30 ± 4 years. Each volunteer performed a series of breath holds for 15 to 20 heartbeats, during which non-contrast-enhanced MRA was performed. The volunteers were instructed to perform the breath holds in a consistent manner with end-expiration chest positions.

### Imaging protocol

All CMR was performed on a GE Optima 450w 1.5 T MR system using a 32-channel cardiac phased-array coil. The coronary artery imaging protocol consisted of 4 steps: (1) Localization: MR-Echo, the stock realtime sequence, was used to find and bookmark the short-axis 2-chamber view, and the long-axis 2-chamber through the left ventricle; (2) Cine CMR gating window calibration: the bookmarked views were used to prescribe a 4-chamber slice for a cine acquisition, from which the diastasis imaging window is determined; (3) MR Septal Scout gating window calibration: from the 4-chamber cine view, a slice is prescribed along the ventricular septum for fast, projection imaging. From this Septal Scout data, the timing of diastasis is also determined; and, (4) High-resolution MRA acquisition: a 4-cm slab is prescribed obliquely from above the aortic valve to the right lateral atrioventricular (AV) groove for 3-dimensional (3D) SSFP imaging. The prescription is intended to cover: the left and right coronary ostia; the entire right coronary artery up to the posterior-lateral branch; the left main (LM), proximal left anterior descending (LAD) and left circumflex (LCx) coronary arteries; and, the associated branches including the proximal conus and sino-atrial (SA) nodal arteries. The latter 3 steps are detailed below.

### Cardiac gating calibration using cine CMR

To determine the optimal data acquisition window relative to the R-peak of the ECG, a long-axis 4-chamber cine was acquired using a breath-held SSFP sequence with an in-plane resolution of 1.4 × 1.8 mm and 5-mm slice thickness (FOV: 35 cm; TR/TE/flip angle: 3.9 ms/1.7 ms/45°; retrospective gating: 30 phases/cardiac cycle; LPS: 16). The cine MR images were cropped by a rectangle that encompassed the 4 chambers of the heart across all frames to reduce the amount of stationary background tissue. The images were exported to MATLAB® and processed using a custom script. Frame-to-frame correlation coefficients (CC) were calculated [8]. The CC function was spline-interpolated and the inflection points (second-derivative nulls) between the E and A wave peaks were used to define the start and end times of the diastasis window (*W*_cine_) as determined by cine-CMR. This method of using inflection points is intended to capture the points of approach toward and departure from a diastasis period that is presumed to (1) exist; and (2) be stationary according to the cine-CMR images. In our experience, compared to threshold methods, this approach is more robust against minor motion variations observed within diastasis.

### Cardiac gating calibration using the MR Septal Scout

The Septal Scout is a high-temporal-resolution motion monitoring sequence for the ventricular septum. In particular, it observes the expansion and compression of the basal interventricular septum (IVS) along the long axis of the heart, which has been found to correlate with global epicardial motion [9]. The high temporal resolution is achieved by foregoing one dimension of spatial encoding. It is similar to a respiratory navigator for the diaphragm except rather than being interleaved with an imaging sequence, it is currently implemented as a pre-scan for calibrating ECG gating.

**Figure 1:**
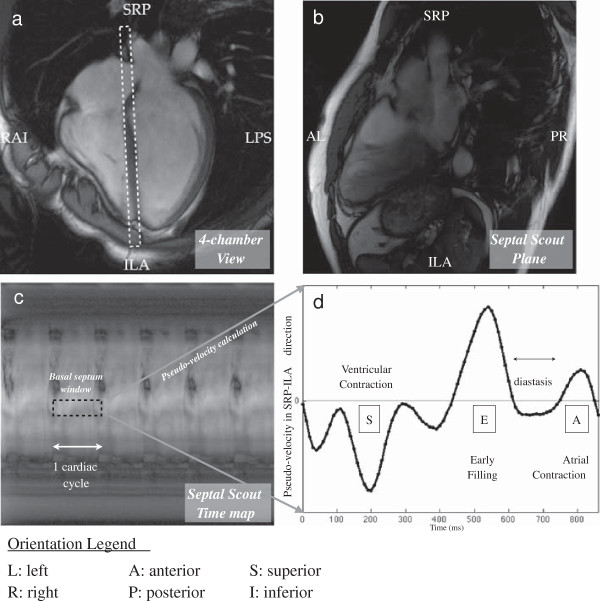
**An illustration of the Septal Scout method.** The Septal Scout monitors long-axis motion of the septum using projection imaging of a slice along the ventricular septum. **(a)** The 4-chamber long-axis view from which the Septal Scout is prescribed is shown. The dashed white rectangle shows the graphical prescription of the Scout Plane, which is perpendicular to the 4-chamber view and runs along the ventricular septum. **(b)** An image of the Scout Plane is shown here for illustration. The Septal Scout does not acquire this image, rather, it acquires much more quickly projections (in the AL-PR direction) of this image plane. **(c)** Successive Septal Scouts are displayed as vertical line images and appended along a horizontal time axis. An ROI (dotted black box) is chosen at a depth near the basal septum spanning one cardiac cycle. The image intensities in this ROI are processed using optical gradients to form a pseudo-velocity graph. **(d)** The pseudo-velocity graph is shown. The diastasis period is visible as a plateau region in between the early filling (E-wave) and atrial contraction (A-wave) cardiac phases.

The Septal Scout is a modified SSFP sequence with phase encodes turned off. It acquires a one-dimensional (1D) projection of the prescribed septal Scout Plane (5 to 10 mm thick) every TR, which may be as short as 3 ms (see Figure 1a). In our experience, a 10-ms TR provides an ample data frame rate and is chosen to permit relatively fast data processing. The rest of the imaging parameters are as follows: flip angle = 55º; FOV = 31 cm; spatial resolution along the scout = 0.8 mm.

Successive Septal Scout projections are appended along a time axis, much like in M-mode ultrasound (see Figure 1c). An ROI spanning a depth ± 2.5 mm is selected to coincide with the location of the basal septum. This ROI height is set arbitrarily to be small enough for the ROI to be considered rigid, and large enough to allow for spatial signal averaging. The signals along the depth dimension (spatial axis) within the ROI are averaged to improve the signal-to-noise ratio (SNR). Along the time axis, the ROI spans at least one cardiac cycle to provide at least one estimate of diastasis timing. This produces, for the basal septum, an intensity plot over time.

To estimate tissue motion, we apply the gradient optical flow method [10] to the intensity plot. This method calculates pixel intensity variations over time to determine object motion at a location. In our case, this is feasible for tracking septal motion because the septum comprises of non-uniform pixel intensities as seen in Figure 1b. Also, this method may be simplified in our application because to identify the timing of diastasis, we only require displacement and velocity indices on a relative rather than an absolute scale. Hence, the ROI intensity plot is treated as a pseudo-displacement function of tissues moving across the basal septum ROI. The pseudo-velocity function is obtained by taking the temporal derivative of the pseudo-displacement plot, which reveals the characteristic E and A waves that border the diastasis period (see Figure 1d). Similar to the processing of the cine-CC function, inflection points between the E and A waves are considered to be the start and end times of the diastasis period.

The Septal Scout acquisition is triggered from the R-peak of the ECG and lasts 5 seconds. Currently, the scout is performed at the beginning and end of a practice 20-second breath hold. In over half of the subjects, heart rate was observed to increase during the course of a 20-second breath hold by 5 to 10 bpm. The intersection of diastases across all heartbeats observed produces the multi-heartbeat diastasis window (*W*_sep_) as determined by the Septal Scout. The two Septal Scout acquisitions at the beginning and end of a breath hold allow us to find a cardiac rest period that is robust across the expected heart rate variation. This is not feasible for the cine acquisition, which needs to acquire data for the full duration of the breath hold.

### Comparison with tissue doppler echocardiography

It has been shown that tissue Doppler echocardiography (TDE) of the IVS was able to identify accurate coronary artery diastasis windows [9]. The first six subjects in this study formed a subcohort for comparing the Septal Scout to the TDE technique with respect to the timing estimation of diastasis windows. TDE of the IVS was performed by an experienced ultrasound technologist in this sub-cohort using a Philips iE33 system with a phased array transducer operating at 3.5 MHz. The IVS was imaged in an apical 4-chamber view at 150 fps; velocity was resolved at ±15 cm/s near the basal septum. Diastasis window estimates were determined from the IVS velocity plots using a custom MATLAB® script. For each velocity plot, similar to the Septal Scout method, the inflection points between the E and A waves were determined to be the start and end of the associated diastasis period, respectively.

### MRCA acquisition

Each volunteer was imaged twice with 3D SSFP, once with the cine-calibrated imaging window, and once with the septal-motion-calibrated window. The parameters for the acquisition were: 3D fat-suppressed SSFP; TR = 3.9 ms; TE = 1.9 ms; flip angle = 55°; FOV = 35 × 35 × 4 cm; resolution = 1.5 × 1.5 × 2.0 mm; slice oversampling = none. The sequence employed a Cartesian trajectory, with an α/2 pre-pulse and 20 dummy cycles to obtain steady state. The number of TRs per heart beat, and thus also the total scan time, varied with the gating window used.

### Qualitative image comparison

Subjective assessment of image quality was performed by two experienced observers, who were blinded to each other’s results and to the technique with which each dataset was acquired. A 5-point Likert scale was used: 0 = not visible; 1 = artery visible with significant blurring of edges; 2 = artery visible with moderate blurring of edges; 3 = artery visible with mild blurring of edges; and, 4 = artery visible with sharply defined edges. Both observers graded, for each 3D dataset, the large vessel segments: proximal RCA, mid RCA, LM, proximal LAD, and prox LCx; and the small vessel segments: 1st acute marginal branch, 1st obtuse marginal branch, 1st diagonal branch, conus artery, and SA nodal artery. The scores were averaged within each vessel size category.

### Quantitative image comparison

To compare the image quality of the two gating calibration methods, we measured the SNR, and vessel sharpness. The SNR was determined by taking the mean signal intensity measured in a region-of-interest (ROI) in the aortic root blood pool and dividing it by the standard deviation of a noise ROI measured outside the patient. The same ROIs were used between the cine-calibrated and Septal-Scout-calibrated scans. Vessel sharpness was determined by full width half maximum (FWHM) measurements that are made using a custom MATLAB® script. For each coronary artery segment, 3 cross-sectional views at 1 mm increments along the vessel were selected. For each view, the inside of the vessel is selected by a user via a mouse click. The centroid is automatically identified as the centre, and 12 radial edge profiles separated by 30° are generated. The FWHMs are calculated and averaged across the radial profiles. In this case, the FWHM serve as both a metric of vessel boundary sharpness, and an estimate of vessel diameter.

### Statistical analysis

Pearson’s correlation coefficient and Bland-Altman analysis were used to compare the start and end times of the diastasis windows as estimated by the Septal Scout and tissue Doppler techniques. General statistics are reported as mean ± standard deviation.

The Wilcoxon signed-ranks test was used to test for differences in all the qualitative as well as quantitative metrics between the Septal Scout and cine-CMR methods for gating window identification. This test is also used to evaluate whether the inter-observer scores are significantly different. All comparisons from the signed-ranks tests are presented as the median [IQR], where IQR is the interquartile range representing the difference between the third and first quartile marks. A two-tailed test with P-values ≤ 0.05 was interpreted to indicate statistical significance.

## Results

### Comparison of the Septal Scout with tissue Doppler echocardiography

**Figure 2:**
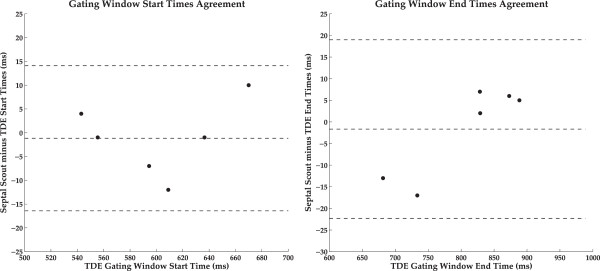
**Comparison of the Septal Scout with tissue Doppler.** Bland-Altman plots showing high agreement of the start and end times (relative to the R-peak on the ECG) of gating windows determined by the TDE and MRI Septal Scout methods.

In the sub-cohort component of this study comparing the Septal Scout to TDE of the IVS, high agreement was observed in the estimation of the start and end times of the diastasis window. The correlation coefficients of the two techniques for both the start and end times were greater than 0.98 (*p* < 0.001). Figure 2 shows the corresponding Bland-Altman plots. We modified the Bland–Altman technique to plot, on the independent axis, the TDE timing data instead of the mean between the two compared data sets. The purpose of this was to reflect the fact that the TDE timing data is in this case the reference.

### Angiography image quality

**Figure 3:**
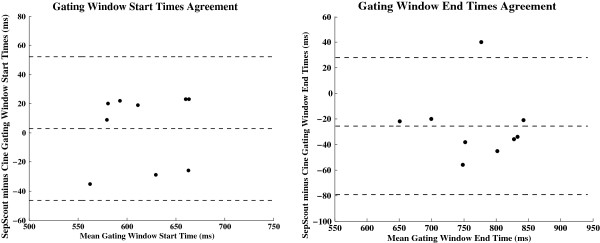
**Comparison of the Septal Scout with cine-CMR.** Bland-Altman plots comparing the start and end times (relative to the R-peak on the ECG) of gating windows determined by the CMR Septal Scout and cine-CMR methods.

MRCA was successfully performed in all subjects. The identified start and end times of the cardiac gating windows by the Septal Scout and cine-CMR techniques are summarized in Table 1; the corresponding Bland-Altman plots are shown in Figure 3. A small amount of heart rate variability (HRV) of typically less than 10 bpm was observed in each subject during the breath holds. In all of the subjects except one, the heart rate was monotonically increasing over the course of the breath hold; the exceptional case experienced a monotonic decrease.

**Table 1:** Septal Scout vs Cine-CMR gating windows

**Subject**	**Heart rate**	Gating window start time* (ms)		Gating window end time* (ms)		**Gating window duration (ms)**	
**#**	**(BPM)**	**Septal scout**	**Cine-CMR**	**Septal scout**	**Cine-CMR**	**Septal scout**	**Cine-CMR**
**1**	56 - 62	621	602	780	825	159	223
**2**	68 - 75	545	580	640	662	95	82
**3**	61 - 67	591	571	690	710	99	139
**4**	55 - 60	672	649	816	850	144	201
**5**	53 - 59	675	652	832	853	157	201
**6**	52 - 62	650	676	810	846	160	170
**7**	59 - 63	584	575	721	777	137	202
8	55 - 50	604	582	797	757	193	175
9	68 - 72	615	644	733	771	118	127

*Start and end times are relative to the R-peak on the ECG.

**Figure 4:**
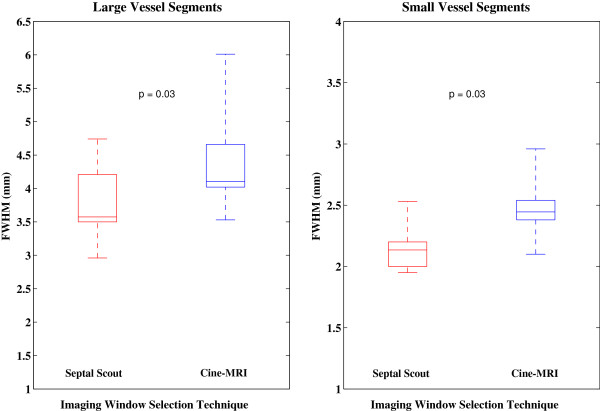
**Full width half maximum measurements of vessel segments.** Box plots of the FWHM measurements of the large and small vessel segments groups comparing the Septal Scout to the cine-CMR technique for selecting the timing parameters of imaging windows.

Vessel segments were sharper when imaged during the Septal Scout gating windows for both the large and small diameter groups (see Figure 4). The large vessel segments were consistently measured to have a smaller FWHM in the Septal Scout group than the cine-CMR group, with a significant difference (*p* = 0.03) between the median widths of 3.6 [0.7] mm versus 4.1 [0.6] mm, respectively. The small vessel segments were also measured to have a significantly (*p* = 0.03) smaller FWHM in the Septal Scout group than in the cine-CMR group, with median widths of 2.1 [0.2] mm versus 2.4 [0.2] mm, respectively.

**Figure 5:**
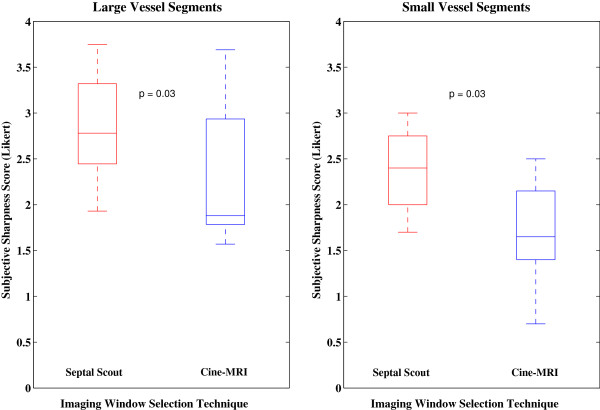
**Observer #1 sharpness scores.** Box plots of the Observer #1 sharpness scores of the large and small vessel segments groups comparing the Septal Scout to the cine-CMR technique.

**Figure 6:**
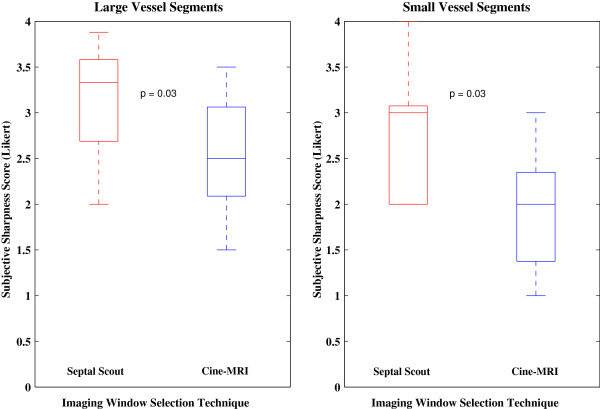
**Observer #2 sharpness scores.** Box plots of the Observer #2 sharpness scores of the large and small vessel segments groups comparing the Septal Scout to the cine-CMR technique.

**Figure 7:**
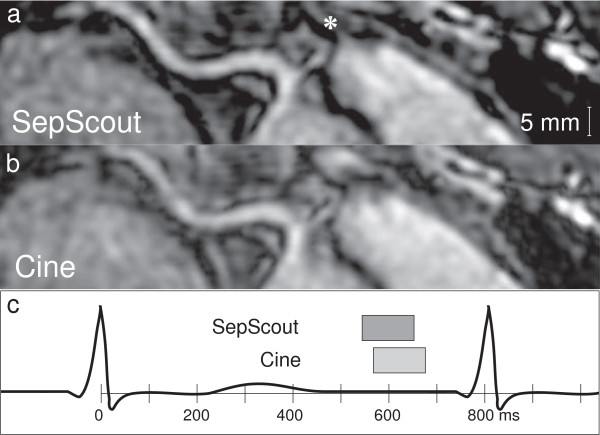
**Image of a proximal right coronary artery.** Image of a proximal RCA segment with a conus branch (*) acquired during **(a)***W*_sep_, and **(b)***W*_cine_. A timing diagram illustrating gating windows of the two techniques is shown in **(c)**.

**Figure 8:**
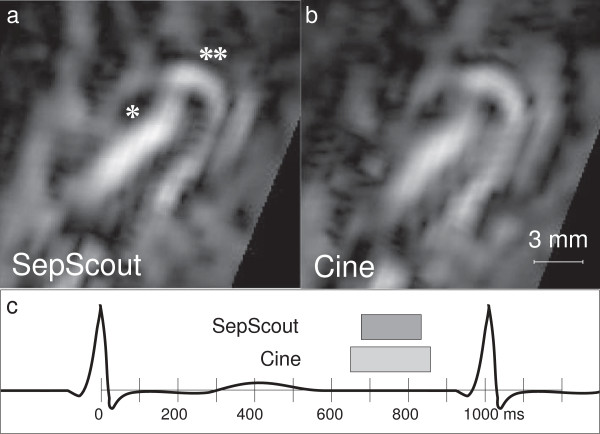
**Image of a conus artery.** Image of a conus artery (**) branching off of the proximal RCA (*) acquired during **(a)***W*_sep_, and **(b)***W*_cine_. A timing diagram illustrating gating windows of the two techniques is shown in **(c)**.

**Figure 9:**
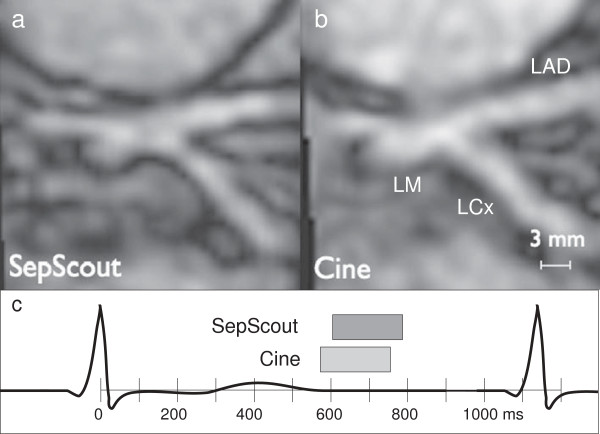
**Image of a left main artery bifurcation.** Image of a left main artery bifurcation acquired during **(a)***W*_sep_, and **(b)***W*_cine_. A timing diagram illustrating gating windows of the two techniques is shown in **(c)**.

Subjective assessment of vessel sharpness also showed an improvement for the Septal Scout-gated scans. Large vessel segments from the Septal Scout group obtained higher sharpness scores from both Observer 1 (2.8 [0.9] vs. 1.9 [1.2], *p* = 0.008; see Figure 5) and Observer 2 (3.3 [0.9] vs. 2.5 [1.0], *p* = 0.008; see Figure 6). Similarly, small vessel segments from the Septal Scout group obtained higher sharpness scores from Observer 1 (2.4 [0.8] vs. 1.7 [0.8], *p* = 0.016; see Figure 5) and Observer 2 (3.0 [1.1] vs. 2.0 [1.0], *p* = 0.016; see Figure 6). The difference between the scores of two observers were not statistically significant (Large vessels: 2.6 [1.2] vs. 3.1 [1.1], *p* = 0.10; small vessels: 2.3 [0.7] vs. 2.4 [1.0], *p* = 0.49). Sample images and the corresponding gating windows are shown in Figures 7, 8, and 9.

There were no significant differences in SNR between the MRCA images acquired with the Septal Scout and cine-CMR techniques (23.5 [12.3] vs. 25.0 [6.6], *p* = 0.18).

## Discussion

In this study, we have successfully applied the MR Septal Scout technique to find more accurate cardiac gating windows than those obtained by cine-CMR. The use of gating windows determined by the Septal Scout led to a significant increase in vessel sharpness in MRCA images.

The principle behind the Septal Scout is to trade off extraneous spatial data, specifically a dimension of spatial encoding, for faster sampling of a targeted component of cardiac motion, specifically, the 1D long-axis motion of the basal ventricular septum. While the comparison of the Septal Scout to the cine-CMR technique is affected by other factors such as (1) imaging tissues in the Septal Scout plane which is perpendicular to the 4-chamber plane, and (2) acquiring 1D vs 2D data, it is believed that the superior temporal resolution of the Septal Scout is the most important factor for its improved performance.

In the subcohort comparison between the Septal Scout and TDE methods, the timing differences of the estimated diastasis periods sometimes reached as high as 15–20 ms. Since the Bland-Altman plots (see Figure 2) reveal no biases, this may reflect inherent variability within the methods.

The Septal Scout technique was superior to the cine-CMR technique in terms of being able to adapt to the HRV that was observed during a breath hold. For cine-CMR, modest HRV may cause incorrect data binning, which in turn may cause motion blurring. In contrast, the Septal Scout essentially freezes cardiac motion within its 10-ms frames. The superior acquisition speed permits the identification of the diastasis window on a per heartbeat basis, and provides a set of gating windows over multiple heartbeats that varies with heart rate. This ability to determine a gating window that is robust across an observed range of HRV during a breath hold is believed to have contributed to the ability of the Septal Scout method to obtain sharper MRCA images in this study.

As a result of the slice excitation, the Scout Plane extends beyond the beating septum to include many unwanted static signal sources such as from the chest and back. The Septal Scout acquisition may alternatively be obtained by the use of 2D RF excitation pulses to further isolate the ventricular septum. For example, a 2D spiral RF pulse [11,12] may be used to excite a cylinder of tissue along the septal wall on the 4-chamber long-axis plane. Note that 2D RF pulses typically require longer durations. This alternative implementation must therefore consider the trade-off between the duration of the excitation pulse, the quality of the excitation profile, and the resultant TR. Generally, sharper excitation profiles require longer excitation pulses. A TR equal to or less than 10 ms should be maintained to provide sufficient temporal resolution for motion tracking. The design specifics of this alternative excitation method are left for future investigation.

The use of a 10-ms TR for the Septal Scout may introduce susceptibility-related artifacts that otherwise may be avoided with a shorter TR. In this study, however, this did not appear to have affected the ability of the Septal Scout to monitor the displacement of the basal septum and identify periods of quiescence. This is likely because the basal septum is a small ROI in a well-shimmed area.

The FWHM assessment of vessel profiles required the reformatting of vessel segments into a cross-sectional orientation. Theoretically, the asymmetric intrinsic resolution of the MRA data (1.5 × 1.5 mm^2^ in-slice; 2 mm through slice) may affect the FWHM comparison if the actual vessel orientations were significantly different between the cine-guided vs. the septal scout-guided acquisitions. For example, if one method produced a gating window during which a vessel segment is oriented much more perpendicular to the slice orientation of the 3D image acquisition, the vessel cross-sections would appear sharper due to the higher in-slice resolution. We did not, however, observe this effect. The intrinsic resolution was near isotropic. And, this effect should not have systematically favoured one method of the study in particular.

The Septal Scout, in general, is sensitive to placement. There are two main types of septal localization errors: vertical and horizontal. Prescribed from a 4-chamber long-axis cine, vertical localization is achieved by aligning the Scout Plane such that it contains the septal wall throughout the cardiac cycle. Missing the septum at any time will result in the loss of the signal of interest. This type of error should be avoided. The prescription of the 4-chamber long-axis plane is the horizontal localization. Deviation by the 4-chamber cine from the horizontal plane of the heart is, however, not a significant concern. This is because the long-axis septal motion will still be the dominant component of motion observed on the Septal Scout. Assuming a good placement, respiratory and general patient motion may still introduce septal localization errors. Currently, this method relies on the use of breath holds to suppress respiratory motion. Like previous authors, this was found to work well for breath holds near or under 20 seconds [13].

In this study, a gradient optical flow calculation [10] was used to determine septal motion from the Septal Scout data. A correlation-based alternative was considered during experimental design using preliminary data. In this approach, a line ROI from a Septal Scout projection was tracked using maximum correlation to a new position in the subsequent projection. The ROI is updated at the new position, and tracked in the subsequent projection, and so on. In our initial experience, the correlation approach was inferior to the optical flow approach because the line ROIs have very few data points to statistically overcome image noise.

In CMR, navigators are 1D signals that monitor the displacement of an edge structure such as the diaphragm during tidal breathing [14]. Navigators are typically performed continuously (on their own and interleaved with imaging) and processed in realtime in conjunction with ECG gating to provide triggers for starting and stopping image data acquisition depending on the navigator position and cardiac phase. The Septal Scout may potentially be adapted into a Septal Navigator. The Septal Navigator function would be a realtime cardiac motion signature; it also may be compatible with a respiratory navigator. In an integrated gating system, the ECG R-peak may be used to trigger contrast preparation and the Septal Navigator may be used to trigger the start and end of imaging per heartbeat. A possible limitation may arise from the Septal Navigator interfering with the image acquisition during diastasis. The Scout Plane excitation may saturate signal sources in coplanar segments of coronary arteries, making them unavailable for imaging. 2D RF pulse excitations may be a solution being less intrusive than a slice excitation. Another issue is that the Septal Navigator may not maintain steady state magnetization, and may require a switch to low-flip-angle spoiled gradient recall methods instead of SSFP. These potential considerations are left for future investigations.

Direct cardiac motion monitoring is a shared concept between the Septal Scout and the anterior left ventricular (LV) wall navigator presented in the past by Stuber et al. [15]. A major distinguishing feature, however, is the location of the Septal Scout versus that of the LV navigator. Different epicardial regions have been known to have different diastasis timings [4,5,16]. The Septal Scout is more suitable for whole-heart angiography; basal septal diastasis is an accurate surrogate for ventricular diastasis [9]. To our knowledge, there has not been work presented on the CMR monitoring of septal motion for determining cardiac gating windows.

The performance of the Septal Scout in the presence of cardiac pathology has yet to be tested. In general, septal wall defects will likely change the relationship between septal and ventricular diastasis. One particularly problematic condition is known as the Septal Bounce, which is a paradoxical bouncing motion of the IVS initially toward and subsequently away from the LV during the beginning of diastole [17]. This condition is typically observed in constrictive cardiomyopathy, and is visible on a 4-chamber long-axis cine CMR. Septal Bounce may cause vertical plane localization errors in the Septal Scout technique. The behaviour of the Septal Scout in the presence of various septal defects should be an investigative component in future patient studies. It is important to know if and when septal diastasis is no longer an accurate surrogate for ventricular diastasis.

In our future work, we intend to explore the use of the Septal Scout to determine the onset of ventricular systole, which is currently detected by the R-peak of the ECG. This provides the benefit of not having to maintain an ECG signal to perform cardiac-gated MR imaging. Currently, the ECG signal may arbitrarily deteriorate due to loosened connections at the chest electrodes; also, R-peak detection may fail due to significant T-wave amplification [18], which may sometimes be a problem even with the use of the vector cardiogram.

## Conclusions

In this chapter, the MR Septal Scout was shown in a healthy volunteer study to be effective at determining accurate cardiac gating windows. Specifically, this new technique is consistently more accurate than the cine-CMR method, the current clinical standard. The improvement in cardiac gating accuracy provided by the Septal Scout lead to sharper coronary MRA images. Furthermore, the Septal Scout was shown to provide diastasis windows that are in close agreement with those provided by TDE measurements of septal motion.

## Abbreviations

1D: One-dimensional; 2D: Two-dimensional; 3D: Three-dimensional; AV: Atrioventricular; CAD: Coronary artery disease; CC: Correlation coefficient; CMR: Cardiovascular magnetic resonance; CMR-CGS: Cardiovascular magnetic resonance based cardiac gating system; ECG: Electrocardiogram; FOV: Field of view; FWHM: Full width half maximum; HRV: Heart rate variability; IQR: Inter-quartile range; IVS: Interventricular septum; LAD: Left anterior descending (artery); LCx: Left circumflex (artery); LM: Left main (artery); LV: Left ventricle; MR: Magnetic resonance; MRA: Magnetic resonance angiography; RCA: Right coronary artery; RF: Radio-frequency; ROI: Region of interest; SNR: Signal to noise ratio; SSFP: Steady state free precession; TDE: Tissue Doppler echocardiography; TE: Echo time; TR: Repetition time; LPS: Lines per segment.

## Competing interests

This work was supported in part by GE Healthcare. The authors declare that there are no other competing interests.

## Authors’ contributions

GL and GAW contributed in the design of the study and drafted the manuscript. GL coordinated the study and performed the statistical analysis. All authors contributed in discussions and approved the final manuscript.
